# To the Editor of the *Dental Register*

**Published:** 1900-10

**Authors:** 


### TO THE EDITOR OF THE "DENTAL REGISTER."

The editor of this journal desires to express his thanks to the editor of the *Dental Register* for his editorial in the July number, in which he undertakes to give the views of the editor of this journal as he, the editor, understood them from reading a leading article in the July number of the International Dental Journal, entitled "Is Dentistry drifting into Medicine?" If the editor of the *Register* failed to understand the underlying thought of the editorial in question, it must certainly be true that the writer failed to clearly convey his meaning to the average reader.

The editor of the International is, however, inclined to think that our friend failed to grasp either the editorial or the facts that it endeavored to elucidate. To sustain his conclusion he quotes one paragraph that "Dental education has been gradually tending towards the medical degree for the past thirty years, and those who have watched the trend of thought have long since come to the conclusion that at no distant period dentistry would be merged into medicine, and that the dentist of the future would practise as a specialist under the one general title of Doctor of Medicine." The editor then follows this with the statement, "A comparison of the curriculum of to-day with that of thirty years ago will show that little has been added of medical technique, while great additions have been made along the line of the medical sciences, scarcely anything in the way of clinical or practical medicine."

The truth of this must be admitted, and directly supports the thought of the writer that this continued advance must certainly and surely lead to the medical degree. There is no escape from the facts of evolutionary progress.

The editor assumes that the writer was so impressed, at the recent meeting of the Section of Stomatology, American Medical Association, with "the profound conclusions of these eminent medical specialists and experienced dental practitioners and educators," that he at once jumped to the conclusion "that the ideal of dental education must eventually be the degree of Doctor of Medicine." Had our friend followed the article carefully he would have found the following: "Educators of experience accept these changes with hesitation and doubt. They cannot avoid the conclusion that the practical side of dentistry has suffered and will continue to suffer in proportion as the curriculum is extended at the top. Like an inverted pyramid it must eventually fall by its own weight." The truth is, the writer was not unduly impressed with the arguments advanced at that meeting, well expressed as they were, but has for years seen, as he stated, that the general trend of dental education has been in the direction of the medical degree.

The question is not one of personal preference. If that were to be the standard of judgment, the writer would decidedly be on the side of continuing dentistry as a distinct profession. In fact, the possibility of dentistry being eventually absorbed into medicine creates a feeling of decided antagonism, but the question is one so broad that it dwarfs mere personality, and necessitates an intelligent outlook and a careful weighing of facts that have led step by step, nearer and nearer, to the point when its consideration will be forced upon the higher dental schools. We simply request our friend and coworker to enlarge his vision, and perhaps, whether willingly or unwillingly, he will reach similar conclusions.

